# Critical Thinking and Motivation in Vocational Training and Baccalaureate: A Comparison Study of Students of Spanish Nationality, Unaccompanied Foreign Minors and Young Care Leavers

**DOI:** 10.3390/ijerph19095272

**Published:** 2022-04-26

**Authors:** Noelia Parejo-Jiménez, Jorge Expósito-López, Ramón Chacón-Cuberos, Eva María Olmedo-Moreno

**Affiliations:** Department of Research Methods and Diagnosis in Education, Faculty of Education, University of Granada, 18071 Granada, Spain; jorgeel@ugr.es (J.E.-L.); rchacon@ugr.es (R.C.-C.); emolmedo@ugr.es (E.M.O.-M.)

**Keywords:** baccalaureate, vocational training, young care leavers, unaccompanied foreign minors, motivation and critical thinking

## Abstract

The present study analyzed academic motivation and its relationship with dispositions towards critical thinking in a sample of 131 students of Spanish nationality and 131 students of foreign nationality (unaccompanied foreign minors and foreign adolescents who had previously experienced the Andalusian care system). For this, an ex-post-facto study was conducted which was descriptive, comparative, and cross-sectional in nature. The main analyses carried out are of a descriptive and inferential nature, the latter to analyze the differences and associations between the variables of critical thinking and motivation (ANOVA test and an structural equations models) in both groups. Main outcomes included the lack of significant differences in dispositions towards critical thinking between Spanish and foreign students, alongside the existence of significant differences in academic motivation between both of these groups. In addition to this, significant differences were found within the group of Spanish nationality students in the dimensions of critical thinking as a function of intrinsic motivation, whilst such differences emerged in the foreign nationality group as a function of intrinsic and extrinsic motivation and amotivation. Finally, within the group of Spanish students, a significant and positive relationship was found between motivation and critical thinking, being positive and non-significant in the foreign national group. In conclusion, it is necessary to improve dispositions towards critical thinking and educational motivation from the educational system for the inclusion of minors and youths in today’s society.

## 1. Introduction

One of the main challenges faced by teachers is adapting their educational practice to promote in-depth learning in their students [1], whilst also allowing students to develop autonomy at a personal and professional level, as well as resilience, empowerment, self-sufficiency, and appropriate goals that lead to better academic outcomes [2]. Further, teaching should enable autonomous and permanent life-long learning that is well aligned to the characteristics of the new knowledge society and the challenges this implies [3]. 

Such a deep learning approach is “based on intrinsic interest in the content being examined, with strategies being born out of this interest and being focused towards optimising understanding and satisfying curiosity” [4] (p. 218). Various studies have highlighted the relationship of this approach with self-regulated learning [5,6]. Self-regulated learning is based on the use of interconnected and contextualized processes to achieve personal goals. These processes involve metacognition, ability, emotional and behavioral processes, and resilience, with self-efficacy giving shape to these aspects [7]. 

In addition, this approach is related with the development of critical thinking with regards to its components and the way in which it is put into practice or delivered in the classroom [8]. Critical thinking is understood as [9] (p. 18):

a metacognitive process that, through purposeful, self-regulatory reflective judgement (i.e., including the sub-skills of analysis, evaluation, and inference) and a disposition towards thinking (i.e., the extent to which an individual is inclined or willing to perform a given thinking skill), increases the chances of producing a logical conclusion to an argument or solution to a problem.

Concern exists around the shortage of programs being put into place to develop critical thinking within secondary and higher education students, with this skill being very rarely worked on explicitly in the classroom. Given this, it is necessary to put specific formative actions into action to promote the development of this skill in students [10]. For this, a set of factors that influence the development of this process should be considered. 

Some studies, conducted in the stages of secondary and higher education, have highlighted a positive relationship between motivational aspects and critical thinking [11,12,13]. Self-determination theory (from here on SDT) states that intrinsic motivation and well-internalized forms of extrinsic motivation predict satisfactory outcomes at different educational levels and in different cultural contexts. Furthermore, these types of motivation are reinforced when the basic psychological needs of autonomy, feelings of success and growth (competence), and the sense of belonging and connection to a group (relatedness) are supported [14,15]. 

This is hugely important when it is considered that, in the present day, “new diversity” is found in classrooms. This refers to an overall set of characteristics related with migration, sexual diversity, gender identities, social, economic, cultural and linguistic differences, skills and abilities, ethnic origin, gender, disabilities, etc., highlighting the huge challenge of meeting the educational needs of all students [16]. In light of this, SDT highlights that:

autonomy support has as its central feature attempting to appreciate and respect the internal frame of reference of the learner (…) is thus a central element in cultural competency—that is, in being able to effectively work with people from diverse backgrounds and value systems, whose frames of reference influence their motivations and valuations [15] (p. 5).

Turning attention to unaccompanied foreign minors, a large number of studies have attempted to define their profiles and characteristics. Based on these studies, it can be concluded that these minors come from Morocco, Algeria, Mali, Nigeria, and the Republic of Guinea, and are aged between 15 and 18 years [17]. These adolescents mainly embark upon their journey to migrate in order to find employment and achieve a better quality of life in Europe [18]. “What most moves them is nothing more than access to any type of employment with the aim of normalizing their legal status in the country” [19] (p. 32). To this, it must be added that these adolescents have academic shortcomings, given that many of them were not previously schooled in their country of origin [20].

In order to achieve this goal, migrant adolescents must receive training to equip them for an appropriate transition into the world of work, in this way, improving their integration in their new country. Nonetheless, when migrant minors and adolescents gain access to the educational system, the majority tend to be extrinsically, instead of intrinsically, motivated towards learning. Some of these students may even be demotivated and may see the training process as a waste of time when it comes to reaching their main goal of obtaining work in the short-term. Given this, the present research team agrees with Hidi and Harackiewicz (2000) [21] that teachers must value the benefits of both types of motivation, with intrinsic and extrinsic aspects both having their part to play. Indeed, working on motivation from its multiple dimensions is the only way to be able to help demotivated students.

Thus, motivation promotes in-depth learning and, consequently, critical thinking, in such a way that favors better student academic performance. For all of this, teaching-learning processes based on supporting individual autonomy play and essential role [22], as outlined by SDT. Further, when migrant minors arrive in Spain they find themselves at an ideal evolutionary point to develop their intrinsic motivation, self-conception, and mastery of tools for the betterment of learning processes. This will help them to develop the self-regulated knowledge required to guide them throughout their academic journey, in their transition towards active life [23] and throughout life in general. 

In consideration of all of that discussed above, the present study provides data on academic motivation and dispositions towards critical thinking in foreign students, unaccompanied foreign minors and leavers of the Andalusian care system, in comparison with those of adolescents of Spanish nationality. A scarcity of research exists on motivation and disposition towards critical thinking in migrant adolescents, meaning that contributions of the present work are highly novel to the research field. 

The present study, therefore, establishes the following three aims: (a) examine dispositions towards critical thinking and academic motivation in Spanish and foreign (unaccompanied foreign minors and foreign care leavers) students; (b) analyze the differences between dispositions towards critical thinking and motivation according to nationality; (c) develop a model of the structural relationships between dispositions towards critical thinking and academic motivation using multi-group analysis with structural equations. 

## 2. Materials and Methods

### 2.1. Participants 

The population included in the present study was composed of secondary education students enrolled on basic vocational training (VT), intermediate VT, and baccalaureate courses, in addition to higher education students undertaking advanced level VT. Both educational levels were being taught at educational centers in the autonomous region of Andalusia (Spain). 

Probabilistic multi-stage sampling was used to select the sample. First, educational centers in the autonomous region of Andalusia were selected through cluster sampling, in accordance with the following criteria: (1) enroll students of foreign nationality who arrived to Spain without a family member of legal guardian, and (2) delivers training courses pertaining to basic VT, intermediate VT, advanced level VT, and baccalaureate education. Second, two groups were established. The first comprised students of Spanish nationality and the second comprised foreign national students. The criteria employed to establish these groups were as follows: (1) both groups were to have similar socio-demographic characteristics, (2) in the case of foreign students, the migrant minor must have arrived to Spain unaccompanied by any family relative and be under the care of the Andalusian care system or, alternatively, be a leaver of this system who had previously received care provision as a minor, (3) foreign students must have been in Spain for more than two and a half years, (4) foreign students must be enrolled as first-time students on basic VT, intermediate level VT, or advanced level VT courses without having received any prior training aside from that granting access to these courses. 

The sample was composed of a total of 262 individuals divided into two groups. The first group was made up of 131 foreign national students, of which 48.09% (*n* = 63) were male and 51.9% (*n* = 68) were female. With regards to the educational level being undertaken, 3.05% (*n* = 4) were enrolled on basic VT, 29.7% on intermediate VT (*n* = 39), 32.06% (*n* = 42) on advanced level VT, and 35.1% (*n* = 46) on baccalaureate studies. The second group was made up of 131 Spanish national students, of which 47.3% (*n* = 62) were male and 52.6% (*n* = 69) were female. With regards to the educational level being undertaken, 6.1% (*n* = 8) were enrolled on basic VT, 32.8% (*n* = 43) on intermediate VT, 34.3% (*n* = 45) on advanced VT, and 26.7% (*n* = 35) on baccalaureate courses. 

### 2.2. Design, Procedure, and Instruments

The present study adhered to an ex post facto design and was descriptive, comparative and cross-sectional in nature. It examined dispositions towards critical thinking and academic motivation in Spanish and foreign national students. To this aim, two instruments were used. 

The first of these instruments pertained to the disposition towards critical thinking scale (DCTS). This scale was conceived by Sosu (2013) [24] and validated for use in the context of the pre-university and university educational setting. Following this, Olmedo-Moreno et al. (2022) [25] validated this scale in the educational context of vocational training, with this version being used in the present study. The scale is composed of 11 items divided into two dimensions, namely, reflective critical thinking—RCT—(items 1, 4, 6, 7, 8, 9, 10, 11) and executive critical thinking—ECT—(items 2, 3, 5). The value of the dimensions is calculated from the arithmetic mean of the scores of the items linked to each dimension. Items are responded to on a Likert type scale with 5 response options, ranging from completely disagree (1) to completely agree (5). In this validation, the scale obtained Cronbach α internal consistency values of 0.745 and McDonald w values of 0.756. 

The second of these instruments, the academic motivation scale, was originally designed and validated in French and in English in a sample of university students [26,27] and, later, adapted into Spanish in secondary education and university students [28,29]. The instrument was recently validated by Olmedo-Moreno et al. (2021) [30] in Spanish in a group of university and vocational training students, with a shortened version being proposed. Expósito-López et al. (2021) [31] also conducted a validation of the tool in Spanish in vocational training and baccalaureate students. This final version of the scale (EMS-SF) is used in the present study. This version is composed of a total of 19 items divided between four dimensions. Namely, these dimensions are intrinsic motivation (items 2, 3, 7, 11, 15, 16), internal extrinsic motivation (items 5, 6, 9, 13, 18, 19), external extrinsic motivation (items 1, 10, 14), and amotivation (4, 8, 12, 17). The value of the dimensions is calculated from the arithmetic mean of the scores of the items linked to each dimension. Items are rated along a Likert type scale with 7 response options, ranging from 1 = “this does not apply to me at all” to 7 = “this completely applies”. This scale obtained an internal consistency value of α = 0.830.

With regards to the procedure applied, first, educational centers were contacted via telephone in order to introduce them to the study and confirm their participation. Following this, a report was sent via email containing study details and the instruments to be administered to students, giving centers the opportunity to read the information carefully. Once this had been done and permission from the educational center had been received, appointments were made for data collection and researchers went out to visit centers. With regards to study participants, data were gathered, making every effort to ensure that all students filled instruments out appropriately. The instrument was administered to the participants from March 2019 to February 2020. It is important to highlight that the present study received approval from the Research Ethics Committee of the University of Granada (1678/CEIH/2020), adhering to all recommendations throughout the process. The ethical requirements of the Declaration of Helsinki [32] were also followed, placing special emphasis on securing informed participant consent and ensuring data anonymity and confidentiality. 

Once instruments had been administered to the sample, data were imported to the data analysis software program IBM SPSS^®^ version 26.0 (IBM Inc., Chicago, IL, USA). Following this, preliminary analysis was conducted to examine the normality of data gathered in relation to the different items pertaining to the scales employed. Analysis was based on asymmetry (A) and kurtosis (K). Next, using the previously established study groups (Spanish and foreign nationality students) as a reference, descriptive statistics were examined by calculating means (X¯), standard deviations (σ), and variance (V) pertaining to the indicators of the DCTS and EMS-SF scale. Differences between both groups were examined through t-test analysis. Finally, differences between variables were analyzed via ANOVA and structural equation modelling (SEM) using IBM^®^ SPSS^®^ Amos version 24.0, applying the maximum likelihood method. This approach was taken to compare the relationships produced between all of the examined factors between the two study groups. 

## 3. Results

With regards to the analysis of normality, dispersion was examined through testing the asymmetry (A) and kurtosis (K) of the different indicators pertaining to the DCTS and EMS-SF scales. With regards to the interpretation of results, a limit of ±3.29 was established in line with recommendations made by Mayers (2013) [33] for samples of more than 100 participants. In the present study, values recorded for DCTS items ranged between A = −2.47 and A = 0.551, and K = −0.485 and K = 0.852. With regards to EMS-SF items, recorded values ranged between A = −2.189 and A = 0.965, and K = −1.384 and K = 1.825. 

Once normality of the data had been examined, differences were established between the responses given by Spanish and foreign students to the different items on the DCTS. Outcomes of this revealed significant differences in relation to two items, first, in relation to the “reflective critical thinking” dimension (*p* = 0.025) and, second, the “executive critical thinking” dimension (*p* = 0.003) (Table 1). From a more general perspective, it could be concluded that higher means were found for dispositions related with the reflective critical thinking dimension in both Spanish nationality students ($\bar{x}$ = 4.10; σ = 0.67) and foreign nationality students ($\bar{x}$ = 4.12; σ = 0.52). With regards to dispositions related with the executive critical thinking dimension, lower means were reported in both Spanish nationality students ($\bar{x}$ = 3.92; σ = 0.75) and foreign nationality students ($\bar{x}$ = 3.86; σ = 0.76). 

Following this, differences between the responses given by Spanish and foreign nationality students were established for the different items of the EMS-SF scale. Outcomes pointed to significant differences in relation to 18 of the 19 items (Table 2). From a more general perspective, it could be concluded that intrinsic motivation was lower in foreign nationality students ($\bar{x}$ = 5.34; σ = 1.29) than in Spanish nationality students ($\bar{x}$ = 5.79; σ = 1.11), with significant differences emerging between both groups (*p* = 0.002). Internal extrinsic motivation was also lower in foreign nationality students ($\bar{x}$ = 5.00; σ = 1.55) than in Spanish nationality students ($\bar{x}$ = 6.34; σ = 0.74), with significant differences emerging between these groups (*p* = 0.000). External extrinsic motivation was also lower in foreign nationality students ($\bar{x}$ = 5.76; σ = 1.21) than in Spanish nationality students ($\bar{x}$ = 6.11; σ = 1.15) with significant differences emerging between the two groups (*p* = 0.016). Finally, amotivation was lower in foreign nationality students ($\bar{x}$ = 2.24; σ = 1.51) than in Spanish nationality students ($\bar{x}$ = 3.71; σ = 1.81), with these differences being statistically significant (*p* = 0.000). 

Next, differences between the reflective and executive dimensions of critical thinking and motivation and amotivation as a function of the groups of Spanish and foreign nationality students were analyzed. It was possible to confirm that, within the Spanish nationality group, significant differences existed in the executive and reflective dimensions of critical thinking as a function of external extrinsic motivation (*p* = 0.030; *p* = 0.003). Differences also emerged in relation to the dimension of executive critical thinking as a function of intrinsic motivation (*p* = 0.012). With regards to the foreign nationality group, significant differences existed in the executive and reflective dimensions of critical thinking as a function of intrinsic motivation (*p* = 0.001; *p* = 0.000) and amotivation (*p* = 0.007; *p* = 0.002), with the executive dimension of critical thinking also showing differences as a function of external extrinsic motivation (*p* = 0.012) (Table 3).

**Figure 1:**
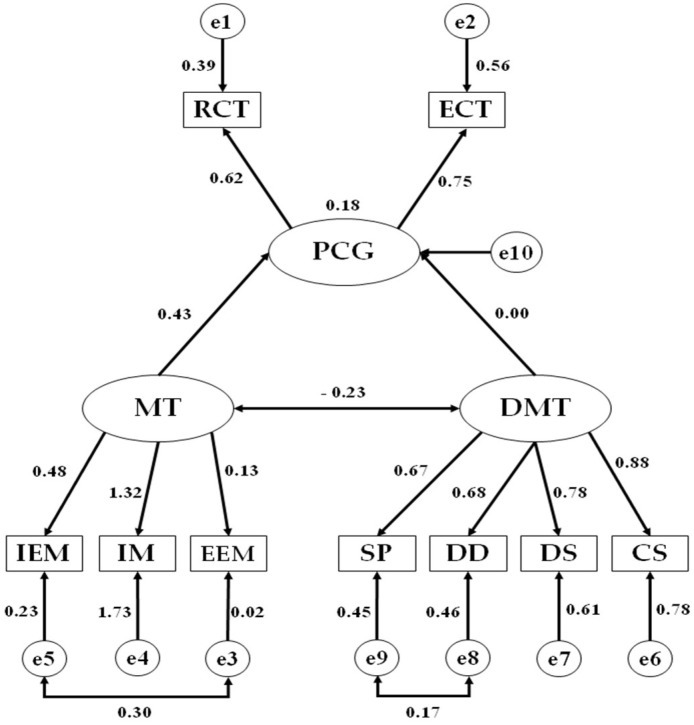
Structural equation model pertaining to students of foreign nationality. PCG, critical thinking; RCT, reflective critical thinking dimension; ECT, executive critical thinking dimension; MT, motivation; IEM, internal extrinsic motivation; IM, intrinsic motivation; EEM, external extrinsic motivation; DMT, amotivation; SP, feeling of wasting one’s time; DD, doubt; DS, lack of interest; CS, lack of meaning.

**Figure 2:**
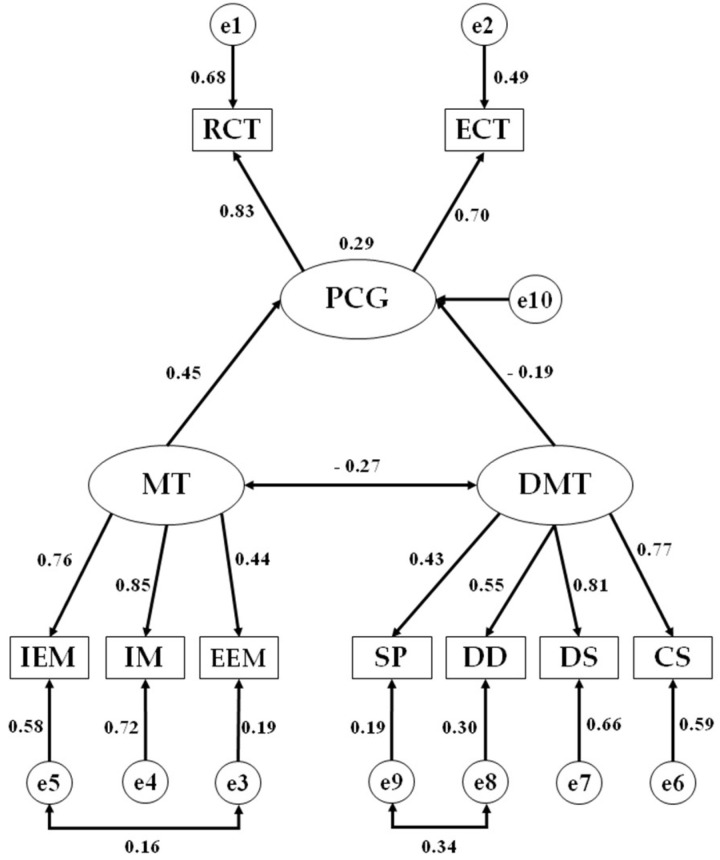
Structural equation model pertaining to students of Spanish nationality. PCG, critical thinking; RCT, reflective critical thinking dimension; ECT, executive critical thinking dimension; MT, motivation; IEM, internal extrinsic motivation; IM, intrinsic motivation; EEM, external extrinsic motivation; DMT, amotivation; SP, feeling of wasting one’s time; DD, doubt; DS, lack of interest; CS, lack of meaning.

Once this analysis had been completed, a structural equation model was developed with the aim of comparing the relationships existing between all of the examined factors according to group assignment (Spanish and foreign nationality students presented in Figure 1 and Figure 2, respectively. The proposed model was composed of two latent or exogenous variables, namely, motivation (M) and amotivation (DMT). Motivation was inferred from the indicators denominating external extrinsic motivation (EEM), internal extrinsic motivation (IEM), and intrinsic motivation (IM). On the other hand, amotivation was inferred from the indicators denominating the feeling of wasting one’s time (SP), doubt (DD), lack of interest (DS), and lack of meaning (CS). These indicators acted as endogenous or observed variables within the proposed model. Furthermore, the endogenous variable of critical thinking (PCG) was included, which was influenced by the exogenous variables of motivation and amotivation (two-way arrows that are interpreted as multivariate coefficients) and whose disposition was inferred through the dimensions of executive critical thinking (ECT) and reflective critical thinking (RCT). Finally, the two-way arrow (covariance) shows the relationship between the exogenous variables of motivation and amotivation, with the error terms being associated with the endogenous variables. 

Good model fit was obtained. The chi-squared value produced was significant (*X*^2^ = 56.648; df = 22; *p* = 0.000), although it is important to highlight that this index cannot be interpreted in a standardized way given its high sensitivity to sample size [34]. For this reason, it was necessary to consider other indices, which were interpreted in line with requisites established by Kock (2014) [35]. To this end, the normative fit index (NFI) was 0.927, with values ≥ 0.90 indicating acceptable model fit. The incremental fit index (IFI) and comparative fit index (CFI) was 0.955 (≥0.95) and 0.954 (≥0.95), respectively, indicating excellent model fit. With regards to root mean square error approximation (RMSEA), a value of 0.075 was obtained, with ≤0.08 indicating acceptable fit. With regards to model parsimony, both the parsimony comparative fit index (PCFI = 0.583 ≥ 0.5) and the parsimony normalized fit index (PNFI = 0.567 ≥ 0.5) were acceptable, according to limits established by Mulaik et al. (1989) [36], which state that values of 0.50 may be acceptable when accompanied by a non-significant X^2^ and goodness of fit indices (NFI, IFI and CFI) of at least 0.90. Further, it is important to highlight that the Hoelter critical N value was 163 when significance was set at 0.05 and 193 when significance was set at 0.01. Thus, it can be concluded that the sample size (*n* = 262) employed in the present study was sufficient for the model fit to be accepted.

Following estimation of standardized regression weights for both the group of Spanish nationality and that of foreign nationality, a significant negative relationship was found between motivation and amotivation in the group of Spanish students (b = −0.268; *p* ≤ 0.05), whilst this relationship was not significant in the group of foreign students (b = −0.230; *p* ≥ 0.05). With regards to the examined indicators and their significant association with these exogenous variables, the indicator that produced the strongest regression weight with regards to motivation in the group of Spanish students pertained to intrinsic motivation (b = 0.851; *p* ≤ 0.001). In contrast, the strongest relationship in the foreign student group pertained to internal extrinsic motivation (b = 0.481; *p* ≤ 0.05). With regards to the indicators that produced that strongest regression weights in relation to amotivation, the strongest indicator in the Spanish student group was lack of interest (b = 0.810; *p* ≤ 0.001), whilst in the foreign student group, the strongest indicator was lack of meaning (b = 0.885; *p* ≤ 0.001) (Table 4). 

Finally, with regards to the relationship of motivation and amotivation with critical thinking, a significant positive relationship was obtained between motivation and critical thinking in the group of Spanish students (b = 0.453; *p* ≤ 0.01). Although a positive relationship was also obtained between these variables in the group of foreign students (b = 0.427; *p* ≥ 0.05), this did not emerge to be significant. With regards to amotivation, a negative but non-significant relationship was obtained (b = −0.191; *p* ≥ 0.05) with critical thinking in the Spanish student group, with virtually no relationship being found (b = 0.002; *p* ≥ 0.05) in the foreign student group. 

## 4. Discussion

In response to the first objective of the research, to analyze the disposition towards critical thinking and motivation, we have obtained results, in general terms, that show both groups of students agree in having dispositions towards reflective critical thinking, although they neither agree nor disagree in having dispositions toward executive critical thinking. With regard to academic motivation, there are significant differences between both groups, with higher motivation in students of Spanish nationality. In response to the second objective, differences between critical thinking and academic motivation based on nationality, we have verified that in the group of students of Spanish nationality there are significant differences in the dimension of executive critical thinking based on intrinsic and external intrinsic motivation; and in the dimension of reflective critical thinking based on external intrinsic motivation. On the other hand, in the group of students of foreign nationality there are significant differences in both dimensions of critical thinking based on intrinsic motivation and demotivation; and in the dimension of executive critical thinking based on external extrinsic motivation. Regarding the third objective of the study, relationships between variables, we show that there is a positive relationship between motivation and critical thinking in both groups, this relationship being significant in the group of students of Spanish nationality.

The deep learning approach, alongside dialogic learning and critical thinking, are important abilities that must be developed by individuals hoping to succeed in the 21st century. Furthermore, development of these abilities goes hand in hand with that of others such as problem solving, being able to learn collaboratively, etc. [37]. Various studies have highlighted the need to promote critical thinking at the levels of secondary education and higher education [10,38,39]. In accordance with this, the Spanish and foreign student groups in the present study coincided in possessing dispositions towards reflective critical thinking. However, neither group stood out with regards to the executive critical thinking dimensions, with no significant differences being found between groups. This suggests that it is necessary to strengthen work in this area within the educational ambit. With regards to academic motivation, it was possible to confirm that significant differences existed between examined groups, specifically, Spanish adolescents compared with foreign unaccompanied minors and young care leavers. Intrinsic motivation, internal extrinsic motivation and external extrinsic motivation was greater in Spanish students. The fact that migrant minors and adolescent care leavers had lower academic motivation is in line with their migratory profile, as discussed previously. Indeed, according to Bravo and Santos-González (2017) [18] and Morales and Parra-González (2020) [19], one of the main aims and priorities of these minors is to find employment as a means to improving the quality of life in Europe. Given this, it is necessary to bring to the attention of these minors that an education that helps them to go down a specific training track is required to promote their inclusion in the job market [40]. In this sense, these minors must be helped to draw up a job insertion project by redefining their life goals in such a way that, whilst they continue to show interest in accessing the job market, they also value the training itself that gives them access to employment [41]. Thus, access to a wide range of training resources, socio-occupation insertion, preparation for emancipation and leading an autonomous life, and legalizing the migrant minor’s status in their country of settlement are all dimensions which must be developed as a priority [42]. 

With regards to levels of academic amotivation, in the present study, both examined groups reported lower levels of amotivation than the different types of motivation, with amotivation being greater amongst Spanish students than migrant students. These findings are not surprising when it is considered that unaccompanied migrant minors go through incredibly difficult experiences on the migration journey, with these having a marked impact on their lives [43]. In this situation, minors are forced to pull themselves together in order to keep moving forwards, turning themselves into more resilient individuals. In this sense they become individuals who “upon being immersed in an adverse situation (…) have the capacity to use protective factors to overcome adversity, grow and develop adequately, ending up maturing into competent human beings, despite an unfavorable prediction” [44] (p. 14). Studies have highlighted the positive relationship between motivation, achievement, and resilience [45,46], making it an important factor to consider when examining motivation and amotivation in these students. Indeed, “resilience is intertwined with education as it is also an affective and collaborative process” [43] (p. 164). This makes it one of the pillars for the promotion of critical thinking and the development of a life project, with the latter being understood as the ability to set a goal and lay out a path towards achieving it [47]. 

In this sense, the present study established differences between motivation and critical thinking in groups of Spanish students and of foreign unaccompanied minors and young care leavers. It was confirmed that significant differences exist among students of Spanish nationality in the dimension of executive critical thinking as a function of intrinsic and external intrinsic motivation. Similar differences emerged in the dimension of reflective critical thinking as a function of external intrinsic motivation. On the other hand, within the group of students of foreign nationality, significant differences existed in the dimensions of critical thinking (reflective and executive) as a function of intrinsic motivation and amotivation, and in the dimension of executive critical thinking as a function of external extrinsic motivation. Furthermore, in the group of Spanish students, a significant positive relationship was confirmed to exist between motivation and critical thinking, whilst a negative but non-significant relationship emerged between amotivation and critical thinking. Similar outcomes were obtained for the migrant student group, with a positive, although non-significant, relationship being found between motivation and critical thinking and an almost non-existent relationship emerging between amotivation and critical thinking. 

These present findings largely agree with those reported previously by other authors in the stages of secondary education and higher education. These prior findings highlighted the positive relationship between motivation and critical thinking [11,12,13,22,48,49]. Special mention should be given to the study conducted by Valenzuela et al. (2011) [50]. This study developed the motivational scale of critical thinking (EMPC), which made it possible to perform an evaluation of the motivation of individuals towards thinking critically. 

Turning attention, more specifically, to the weights calculated in the two groups examined in the present study for each motivational type in relation to general motivation, it could be seen that intrinsic motivation exerted the greatest influence within the Spanish student group. Nonetheless, internal extrinsic motivation exerted the greatest influence on general motivation within the group of young migrants, followed by external extrinsic motivation. With regards to amotivation, the indicator producing the greatest regression weight in the Spanish student group was lack of interest, with lack of meaning being the main indicator in the migrant group. 

These outcomes are not surprising when the profile of unaccompanied foreign minors is considered, as revealed by a number of previously conducted studies [17,51,52]. Generally speaking, these minors have barely received any schooling and have little training. They come from poor families and a non-industrialized context in which they barely have enough resources to survive together with all of their family. Within this context, three different groups can be established. The first group comprises migrant minors who desperately want work and to legalize their status in their country of settlement, with this aim often being reinforced by their own family. The second group is formed by minors who do not have a clear migratory project and, therefore, do not know what will happen in the future. These youth tend to come from unstructured and poor families where they have experienced violence and had a punctuated schooling period marked by authority and violence, often leading them to reject formalized schooling in the country of settlement. The third group is comprised by migrant minors with a profile characterized by crime, aggression and/or health problems, sometimes mental disorders, all of which means they require specialized attention [52]. In view of these characteristics and those already presented throughout the paper, it is logical that migrant students have greater extrinsic motivation and amotivation marked by the view that education lacks meaning given that it competes with other basic needs that need to be met first.

Maslow has already referred to this in his studies on motivation. These studies highlighted that individual motivation depends on individual needs and that only through satisfying these primary (physiological, security, social/belonging) and secondary (recognition and esteem) needs can self-realization be achieved [53,54]. This latter construct is pursued by individuals with greater intrinsic motivation [54]. To a certain extent, this is also underlined by the SDT, which highlights that motivations are reinforced when they help meet the basic psychological needs of autonomy, competence, and relatedness in students [14,15]. 

Intrinsic motivation is related with a deep learning approach [54]. In the same sense, González-Benito et al. (2021) [55] outlined in their study the need to promote feelings of self-efficacy in students through education. The aim of this is to positively influence their performance and intrinsic motivation, with the latter being mainly characterized by the desire to learn. 

As indicated at the beginning of the study, barely any research into academic motivation and dispositions towards critical thinking in unaccompanied migrant students exists. Further, Boada and Casas (2010) [56] highlighted a degree of statistical invisibility in which young people in care find themselves, specifically, related with their education, is accompanied by a scarcity of research and programs orientated towards the inclusion of these young people in the different levels of education (p. 117). 

For this reason, it is hugely important to continue working in this line of research. Thus, as future lines of research, motivation and critical thinking should continue to be examined in unaccompanied foreign minors and young care leavers in comparison with Spanish nationals, as a function of other variables such as sex, age, educational level, prior work experience, etc. Larger-scale studies should also be proposed with the participation of other countries to obtain a more global vision of the subject under study. Finally, as a main limitation, we point out the design of the study itself, which has a descriptive and cross-sectional nature that does not allow establishing causal relationships between the variables studied. However, the multigroup analysis through the structural equation model contributes to alleviate this limitation. In addition, the sample may be too small and contextualized. This is due to the fact that the population of unaccompanied foreign minors and leavers of the Andalusian care system, who are enrolled in vocational training and baccalaureate, is not very large, as well as the difficulty of accessing the sample.

## 5. Conclusions

In the present study, both Spanish nationality students and a group of unaccompanied foreign minors and young care leavers showed strong dispositions towards reflective critical thinking. On the other hand, strong outcomes were not produced in relation to the dimension of executive critical thinking as no significant differences were found between the two groups.

With regards to academic motivation, it was possible to reveal that significant differences did exist between both groups. Motivation was higher in students of Spanish nationality. Within the group of Spanish students, internal extrinsic motivation was the most highly reported motivational type, followed by external extrinsic motivation and, finally, intrinsic motivation. In the case of migrant students, external extrinsic motivation was the most highly reported motivational type, followed by intrinsic motivation and, finally, internal extrinsic motivation. With regards to levels of academic amotivation, levels were generally lower than for motivation in both groups, with amotivation being higher in Spanish students than in migrant adolescents. 

In addition, within the group of students of Spanish nationality, it was confirmed that significant differences existed in relation to the dimension of executive critical thinking as a function of intrinsic and externally regulated intrinsic motivation, with differences also emerging in relation to the reflective critical thinking dimension as a function of external intrinsic motivation. On the other hand, within the group of students of foreign nationality, significant differences existed in the dimensions of critical thinking (reflective and executive) as a function of intrinsic motivation and amotivation, with executive critical thinking also being impacted by external extrinsic motivation. Finally, within the group of Spanish students, it was confirmed that a significant positive relationship existed between motivation and critical thinking, whilst the relationship between amotivation and critical thinking was negative and non-significant. Similar outcomes were obtained in the migrant student group, unveiling a positive yet non-significant relationship between motivation and critical thinking, alongside a barely existent relationship between amotivation and critical thinking.

## Figures and Tables

**Table 1:** Differences between Spanish and foreign nationality students in dispositions towards critical thinking.

Items	NAT.	$\bar{x}$	σ	V	Sig.	D/(r)
RCT11. I often reflect on my actions to see if I can improve them	S	4.21	0.99	0.99	0.485	-
	F	4.12	0.94	0.89		
RCT8. I often think about my experiences to be able to learn from them	S	4.34	0.84	0.71	0.303	-
	F	4.22	0.94	0.89		
RCT9. I tend to check that information is correct before forming an opinion on it	S	3.96	1.13	1.28	0.457	-
	F	4.06	1.02	1.04		
RCT6. It is important to understand the viewpoint of other people about an issue	S	4.50	1	1	0.114	-
	F	4.67	0.71	0.51		
RCT.10. I normally think about the consequences of a decision before acting	S	3.55	1.27	1.61	0.025 *	0.28/0.14
	F	3.89	1.13	1.28		
RCT1.During a debate I think about what it has brought up	S	3.95	1.13	1.29	0.823	-
	F	3.92	1.06	1.13		
RCT7. It is important to justify the decisions I make	S	4.21	1.09	1.19	0.067	-
	F	3.96	1.05	1.11		
ECT4. I often search for new ideas	S	4.02	1	1	0.003 **	0.37/0.18
	F	3.61	1.18	1.41		
ECT2. I often think up new ideas to improve the way I do things	S	4.09	1.02	1.05	0.811	-
	F	4.06	1.04	1.08		
ECT3. I use more than one source or document (internet, books…) to find information for myself.	S	3.95	1.20	1.45	0.636	-
	F	4.02	1.14	1.30		
ECT5. Sometimes I find ideas that contradict my opinion	S	3.66	1.16	1.35	0.412	-
	F	3.77	1.09	1.19		

Note: $\bar{x}$, mean; σ, standard deviation; V: variance; RCT, reflective critical thinking dimension; ECT, executive critical thinking dimension; NAT: Nationality; S: Spanish; F: Foreign; D: d of Cohen; (r): Effect size (r); Sig. *=* * Statistically significant differences at the level of *p* < 0.05, ** Statistically significant differences at the level of *p* < 0.01.

**Table 2:** Differences between Spanish and foreign nationality students in academic motivation.

Items	NAT.	$\bar{x}$	σ	V	Sig.	D/(r)
IM-2. Because I feel pleasure and satisfaction when I learn new things	S	6.24	1.18	1.41	0.001 ***	0.42/0.20
	F	5.69	1.42	2.02		
IM-3. Because I really like going to class	S	4.93	1.78	3.17	0.017 *	0.29/0.14
	F	4.38	1.91	3.65		
IM-7. For the pleasure I feel when I discover new things which I had never seen before	S	6.09	1.53	2.34	0.016 *	0.29/0.14
	F	5.63	1.59	2.52		
IM-11. For the pleasure I feel when broadening my knowledge about topics that interest me	S	6.24	1.30	1.70	0.001 ***	0.40/0.19
	F	5.66	1.56	2.45		
IM-15. Because my studies enable me to continue learning many things that interest me.	S	6.12	1.56	2.46	0.001 ***	0.39/0.19
	F	5.47	1.69	2.88		
IM-16. Because it pushes me to read about topics that interest me	S	5.18	2.26	5.13	0.857	-
	F	5.22	1.81	3.28		
IEM-5. For the pleasure I feel when I better myself in my studies	S	5.78	1.74	3.03	0.000 ***	0.54/0.26
	F	4.78	1.93	3.74		
IEM-6. In order to demonstrate to myself that I am capable of finishing my baccalaureate/course	S	6.20	1.43	2.06	0.000 ***	0.66/0.31
	F	4.98	2.17	4.73		
IEM-9. Because when I perform tasks well in class I feel important	S	5.83	1.64	2.71	0.000 ***	0.63/0.30
	F	4.63	2.11	4.48		
IEM-13. For the satisfaction I feel when I make progress on difficult academic activities	S	6.29	1.23	1.53	0.012 **	0.60/0.29
	F	5.38	1.73	3.02		
IEM-18. Because classes lead to personal satisfaction when I strive to get the most out of my studies	S	7	0	0	0.000 ***	1.62/0.63
	F	4.86	1.86	3.48		
IEM-19. Because I want to demonstrate to myself that I can complete my studies.	S	7	0	0	0.000 ***	1.16/0.50
	F	5.42	1.92	3.72		
EEM-1. Because I need, at least, a baccalaureate/vocational training qualification to find a well-paid job	S	6.07	1.42	2.03	0.037 *	0.26/0.12
	F	5.71	1.33	1.79		
EEM-10. Because I want to “live well” once I finish my studies	S	6.28	1.41	1.98	0.023 *	0.28/0.13
	F	5.87	1.51	2.28		
EEM-14. In order to be able to get a better salary	S	6.01	1.36	1.86	0.000 ***	0.19/0.09
	F	5.72	1.64	2.71		
D-4. I honestly do not know, I think I am wasting my time at college.	S	3.02	2.30	5.30	0.006 **	0.34/0.17
	F	2.29	1.89	3.57		
D-8. I used to have good reasons for going to college but not I ask myself if it is worth the effort going on	S	3.79	2.40	5.76	0.001 ***	0.42/0.20
	F	2.82	2.16	4.68		
D-12. I do not know why I go to college and, honestly, I do not care.	S	3.73	2.41	5.84	0.000 ***	0.87/0.39
	F	1.93	1.65	2.74		
D-17. I don’t know, I don’t understand what I do at college	S	4.32	2.37	5.65	0.000 ***	1.18/0.50
	F	1.92	1.62	2.62		

Note: $\bar{x}$, mean; σ: standard deviation; V: variance; MI: intrinsic motivation; MEI: internal extrinsic motivation; MEE: external extrinsic motivation; D: amotivation; NAT: Nationality; S: Spanish; F: Foreign; D: d of Cohen; (r): Effect size (r); Sig. = * Statistically significant difference at the level *p* < 0.05, ** Statistically significant differences at the level *p* < 0.01, *** Statistically significant differences at the level *p* < 0.001.

**Table 3:** Differences between the dimensions of critical thinking, motivation, and amotivation according to whether students were Spanish or foreign nationals.

Dimensions of Critical Thinking According to Motivation and Amotivation		Spanish Nationality Students		Foreign Nationality Students	
		F	Sig.	F	Sig.
ECT	EEM	1.359	0.180	2.181	0.012 *
	IM	1.951	0.012 *	2.398	0.001 ***
	IEM	1.829	0.030 *	1.320	0.153
	DMT	0.675	0.861	2.199	0.007 **
RCT	EEM	1.434	0.143	1.590	0.092
	IM	1.213	0.250	2.495	0.000 ***
	IEM	2.372	0.003 **	1.384	0.117
	DMT	1.089	0.369	2.447	0.002 **

Note: RCT, reflective critical thinking dimension; ECT, executive critical thinking dimension; IM: intrinsic motivation; IEM: internal extrinsic motivation; EEM: external extrinsic motivation; DMT: amotivation, Fisher F; Sig = * Statistically significant differences at the level *p* < 0.05, ** Statistically significant differences at the level *p* < 0.01, *** Statistically significant differences at the level *p* < 0.001.

**Table 4:** Structural equation models: multi-group analysis stratified according to students of Spanish and foreign nationality.

Association between Variables	RW			SRW
	Estimation	SE	CR	Estimation
Raw and standardized regression weights produced in Spanish students				
PCG ← MT	0.498	0.162	3.079	0.453 **
PCG ← DMT	−0.059	0.034	−1.727	−0.191
RCT ← PCG	1.000	-	-	0.827 ***
EEM ← MT	1.000	-	-	0.441 ***
IM ← MT	1.856	0.507	3.662	0.851 ***
IEM ← MT	1.103	0.234	4.723	0.759 ***
CS ← DMT	1.000	-	-	0.771 ***
DS ← DMT	1.068	0.168	6.376	0.810 ***
DD ← DMT	0.718	0.132	5.429	0.548 ***
SP ← DMT	0.544	0.126	4.326	0.432 ***
ECT ← PCG	0.947	0.208	4.555	0.701 ***
MT ↔ DMT	−0.249	0.121	−2.063	−0.268 *
Raw and standardized regression weights produced in foreign students				
PCG ← MT	0.908	0.503	1.805	0.427
PCG ← DMT	0.000	0.027	0.018	0.002
RCT ← PCG	1.000	-	-	0.623 ***
CS ← DMT	1.000	-	-	0.885 ***
DS ← DMT	0.903	0.097	9.328	0.782 ***
DD ← DMT	1.029	0.130	7.893	0.681 ***
SP ← DMT	0.886	0.114	7.755	0.671 ***
ECT ← PCG	1.733	0.389	4.456	0.747 ***
EEM ← MT	1.000	-	-	0.128 ***
IM ← MT	11.005	6.779	1.623	1.316
IEM ← MT	4.802	2.294	2.094	0.481 *
MT **↔** DMT	−0.051	0.037	−1.386	−0.230

Note: RW, regression weight; SRW, standardized regression weight; SE, standard error; CR, critical ratio; PCG, critical thinking; RCT, reflective critical thinking dimension; ECT, executive critical thinking dimension; MT, motivation; IEM, internal extrinsic motivation; IM, intrinsic motivation; EMM, external extrinsic motivation; DMT, amotivation; SP, feeling of wasting one’s time; DD, doubt; DS, lack of interest; CS, lack of meaning; * Statistically significant differences at the level *p* < 0.05; ** Statistically significant differences at the level *p* < 0.01; *** Statistically significant differences at the level *p* < 0.001; ← Relationships between latent and observed variables; ↔ relationships between exogenous variables.

## Data Availability

Not applicable.
